# A comparison of microbial composition under three tree ecosystems using the stochastic process and network complexity approaches

**DOI:** 10.3389/fmicb.2022.1018077

**Published:** 2022-10-10

**Authors:** Peng Kang, Yaqing Pan, Pan Yang, Jinpeng Hu, Tongli Zhao, Yaqi Zhang, Xiaodong Ding, Xingfu Yan

**Affiliations:** ^1^School of Biological Science and Engineering, North Minzu University, Yinchuan, Ningxia, China; ^2^Key Laboratory of Ecological Protection of Agro-pastoral Ecotones in the Yellow River Basin, National Ethnic Affairs Commission of the People’s Republic of China, Yinchuan, Ningxia, China; ^3^Innovation Team for Genetic Improvement of Economic Forests, North Minzu University, Yinchuan, Ningxia, China; ^4^Shapotou Desert Research and Experiment Station, Northwest Institute of Eco-Environment and Resources, Chinese Academy of Sciences, Lanzhou, Gansu, China

**Keywords:** soil microbial community, assembly patterns, stochastic ecological processes, co-occurrence network, different tree ecosystems

## Abstract

Soil microbes act as “players” in regulating biogeochemical cycles, whereas environmental heterogeneity drives microbial community assembly patterns and is influenced by stochastic and deterministic ecological processes. Currently, the limited understanding of soil microbial community assembly patterns and interactions under temperate forest stand differences pose a challenge in studying the soil microbial involvement during the succession from coniferous to broad-leaved forests. This study investigated the changes in soil bacterial and fungal community diversity and community structure at the regional scale and identified the pathways influencing soil microbial assembly patterns and their interactions. The results showed that broad-leaved forest cover in temperate forests significantly increased soil pH, and effectively increased soil water content, total carbon (TC), total nitrogen (TN), and total phosphorus (TP) contents. Both soil bacterial and fungal alpha diversity indices were correlated with soil physicochemical properties, especially in broad-leaved forest. The bacterial and fungal community composition of coniferous forest was dominated by deterministic process (bacteria: 69.4%; fungi: 88.9%), while the bacterial community composition of broad-leaved forest was dominated by stochastic process (77.8%) and the fungal community composition was dominated by deterministic process (52.8%). Proteobacteria, Acidobacteriota, Actinobacteriota, and Verrucomicrobiota were the dominant phyla of soil bacterial communities in temperate forests. Whereas Ascomycota, Mortierellomycota, Basidiomycota, and Rozellomycota were the dominant phyla of soil fungal communities in temperate forests. Most members of dominant phylum were regulated by soil physical and chemical properties. In addition, the succession from temperate coniferous forest to broad-leaved forest was conducive to maintaining the complex network of soil bacteria and fungi, and the top 20 degree of the major taxa in the network reflected the positive response of microbial interactions to the changes of soil nutrients during forest succession. This study not only shows the mechanism by which species differences in temperate forests of northern China affect soil microbial community assembly processes, but also further emphasizes the importance of the soil microbiome as a key ecosystem factor through co-occurrence network analysis.

## Introduction

Soil microorganisms are important in regulating soil ecological processes and biogeochemical cycles (Zhu et al., 2020; Starke et al., 2021). Heterogeneity in host preference and the environmental adaptation of microorganisms leads to differences in microbial diversity and community composition. Moreover, the microbial assembly patterns are influenced by stochastic (dispersal limitation, homogenizing dispersal, and drift) and deterministic processes (heterogeneous and homogeneous selection (Stegen et al., 2013; Feng et al., 2018). During the secondary succession of forest ecosystems, the assembly of soil microbial communities is dominated by deterministic processes in the early stage and stochastic processes in the later stage (Liu et al., 2021). In addition, soil depth (Luan et al., 2020), elevation gradient (Siles and Margesin, 2016) and other factors also shaped the stochastic and deterministic processes of different soil microbial communities. More directly, plant species differences are important drivers of soil microbial community assembly, and these differences are related to the imbalance of litter input to soil nutrients, such as pH, TC and TN (Kane et al., 2020; Luan et al., 2020). Many studies have revealed the assembly patterns and driving factors of microbial communities in grassland (Kang et al., 2022), agricultural (Jiao et al., 2022) and river (Chen et al., 2019) ecosystems. However, it remains unclear how stochastic and deterministic ecological processes affect the construction of soil microbial communities in the context of stand differences.

In addition, soil microbial communities have complex connections, and their interactions can be described as negative (e.g., competition) or positive (e.g., cooperation) links that share limited resources, where the strength of the interactions largely determines the stability of the microbial community and further influences ecosystem functions (Stegen et al., 2015). Soil microorganisms are very sensitive to environmental changes during the long-term succession of forest ecosystems. For example, the number of nodes and edges of soil microbial co-occurrence network gradually decreased with stand age, and the relationship between species gradually weakened (Bi et al., 2021). And mixed forests tend to have more complex and stable network relationships than broad-leaved and coniferous forests (Chang et al., 2022). It can be seen that soil nutrients play a decisive role in the process of forest degradation and succession or ecological restoration (Liu et al., 2019). Therefore, studying the relationships between network modules and ecological variables can provides meaningful insights into microbial interactions, while also helping to deepen the understanding of soil microbial interactions in the context of stand differences.

Since the 1980s, a series of ecological restoration projects has been implemented in northern China (Zhang et al., 2016; Yang, 2017). *Larix principis-rupprechtii*, which has strong adaptability and high-stress resistance, plays an essential role in the succession of secondary forests in northern China (Zhu et al., 2008). However, during the conversion of natural forests to coniferous forests, the slow decomposition rate of coniferous litter causes an imbalance between nutrient input and output in forest ecosystems, adversely affecting the soil chemical properties and nutrient availability of forests (Yang et al., 2013). Since coniferous and broadleaf forests (two distinct vegetation type divisions of forests in terms of flora) exhibit contrasting characteristics in terms of litter quality and growth strategies (carbon and nitrogen ratio and water and nutrient use efficiency) under global environmental change (You et al., 2014). Therefore, it is particularly important to reveal the assembly patterns and microbial interaction mechanisms of soil microbial communities under this background.

In view of this, this study was conducted in the Liupanshan National Nature Reserve in the northwest of the Loess Plateau in China, where *L. principis-rupprechtii* have been planted for more than 40 years since the establishment of the Liupanshan National Nature Reserve in 1982. Based on the analysis of soil physicochemical properties and microbial community structure during the succession from coniferous forest to broad-leaved forest in Liupanshan National Nature Reserve, the following scientific hypotheses were put forward: (1) The community assembly of soil bacteria and fungi under forest stand differences was influenced by both stochastic and deterministic ecological processes. (2) The conversion of coniferous forests to mixed forests changes the network relationships of soil microbial communities.

## Materials and methods

### Study site and sample collection

**Figure 1:**
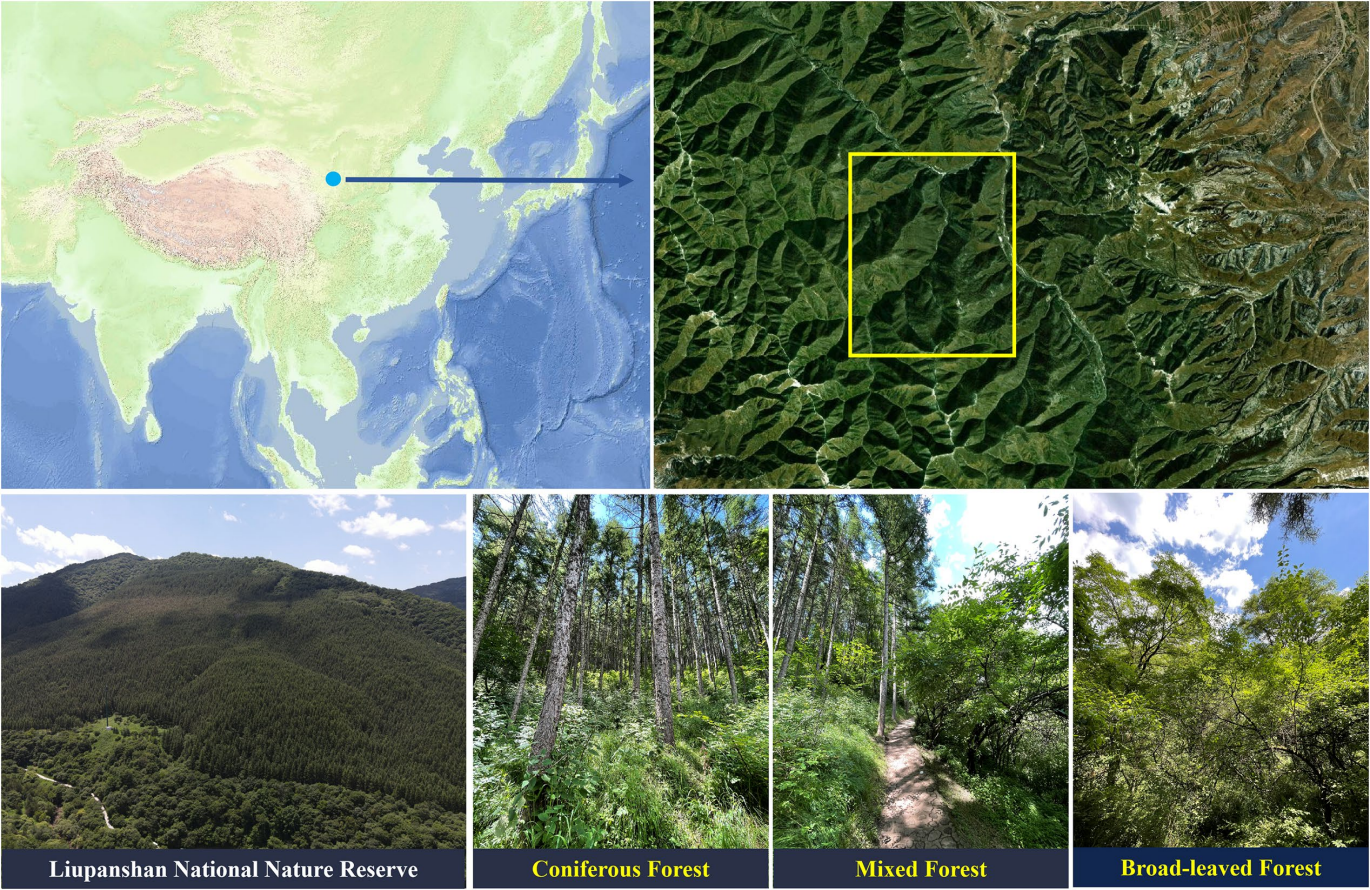
Distribution of sampling sites in Liupanshan National Nature Reserve within Northwestern China. *CF*, coniferous forest; MF, mixed forest; BF, broad-leaved forest.

The study area is located in the Liupanshan National Nature Reserve (106°09′-106°30′E, 35°15′-35°41′N), which is in the successional zone of the warm-temperate semi-humid and semi-arid region in northern China, with high temperatures and rain in summer and dry and cold in winter. The average elevation temperature is 17.4°C, and the average precipitation is 676 mm. The coniferous forests in the region are mainly *L. principis-rupprechtii,* and the broad-leaved forests are mainly *Quercus wutaishanica*, *Betula platyphylla*, *Betula albo-sinensis*, and *Populus davidiana*. In the nature reserve, nine replicate plots of 20 × 20 m (27 in total) were designated as coniferous forest (*CF*) (9 plots), coniferous and broad-leaved mixed forest (MF) (9 plots) and broad-leaved forest (BF) (9 plots) at similar elevations, and the distance between adjacent plots was greater than 200 m (Figure 1). According to the sampling method of Pan et al. (2021), soil samples from each plot were collected by 5-point sampling method after removing litter from the surface. Plant roots were avoided during sampling and soil samples were collected at a depth of 10–20 cm. Five soil samples were mixed and defined as the soil sample of one plot, which was then sieved with 2 mm and divided into two parts. One part was stored in dry ice to extract soil nucleic acids, and the other was used for soil physicochemical characterization (Lin et al., 2021).

### Characterization of physicochemical properties

Soil pH was measured by pH meter (Mettler S220, Mettler Toledo Solutions, Greifensee, Switzerland) (Wang et al., 2019). And soil water content (SWC) was determined using the weighing method before and after drying at 105°C (Bao, 2000). After the 27 collected soil samples were air-dried, the soil total carbon (TC), total nitrogen (TN), and total phosphorus (TP) were determined using atomic absorption spectrometry (iCE 3,500, Thermo Fisher Scientific, Waltham, MA, United States) (Bettinelli et al., 2000).

### DNA extraction and PCR amplification

The total genomic DNA from 27 soil samples was extracted by CTAB method according to the manufacturer’s instructions. The quality and quantity of the extracted DNA were measured using a NanoDrop™ 2000 spectrophotometer (Thermo Fisher, United States) and 1% agarose gel electrophoresis, respectively (Lian and Xing, 2017). The soil microbial community was examined using Illumina NovaSeq 6,000 sequencing analysis (Illumina, San Diego, CA, United States). The Illumina NovaSeq 6,000 sequencing libraries for bacteria were prepared *via* PCR amplification of the V3-V4 hypervariable regions of the bacterial 16S rRNA gene using the primers 338F (5’-ACTCCTACGGGAGGCAGCA-3′) and 806R (5’-GGACTACNNGGGTATCTAAT-3′) with the GeneAmp® 9,700 PCR thermocycler (Applied Biosystems, CA, United States) (Caporaso et al., 2012). The Illumina MiSeq sequencing libraries for fungi were prepared *via* PCR amplification of the internal transcribed spacer (ITS) region of fungi using the primers, ITS5 (5’-GGAAGTAAAAGTCGTAACAAGG-3′) and ITS2 (5’-GCTGCGTTCTTCATCGATGC-3′) (Schmidt et al., 2012). The thermal cycling conditions consisted of an initial denaturation at 95°C for 3 min followed by 27 cycles of denaturation at 95°C for 30 s, annealing at 55°C for 30 s, and extension at 72°C for 45 s, followed by a final extension at 72°C for 10 min (Haas et al., 2011). The purified amplicons were pooled in equimolar concentrations and paired-end sequenced on Illumina platform according to the standard protocols of Novogene Bioinformatics Technology Co. Ltd. (Beijing, China).

### Sequence processing and statistical analysis

Paired-end (PE) library were constructed using a NEXTflex Rapid DNA-SEQ Kit (Bioscience, South San Francisco, CA, United States), and an Illumina NovaSeq 6,000 platform was used for sequencing. Trimmomatic software (Illumina) was used for quality control of Illumina MiSeq sequencing original sequences. FLASH (1.2.11)^1^ software was used for stitching (Magoč and Salzberg, 2011). UPARSE 7.1 software was used for amplicon sequence variants and operational taxonomic units (OTU) clustering analysis (similarity 97%), and UCHIME software was used to remove chimeras (Caporaso et al., 2012). Each sequence was annotated for species classification by the ribosomal database project classifier and compared with the Silva database (SSU128) at a confidence threshold of 0.7 (Quast et al., 2013).

The QIIME program (1.9) was used to calculate the alpha diversity indices (Shannon, and ACE) (Kuczynski et al., 2012). The “linkET”^2^ package in R software (Version 4.1.0) was used to calculate and visualize the correlation between soil physicochemical properties and soil bacterial alpha diversity. A non-metric multidimensional scale (NMDS) based on the Bray-Curtis distance matrix and ANOSIM test with 9,999 permutations were used to illustrate beta diversity (Ziegler et al., 2017). Differences in community composition between sample groups were analyzed using the “vegan” package, and the “Hmisc” and “minpack.lm” package in R software were used to construct the neutral community model (NCM) of bacterial and fungal communities (Chen et al., 2019). The “NST” package in R software was calculated the βNTI and RC values for bacterial and fungal community. The soil physicochemical properties and soil bacterial and fungal communities (Spearman correlation) were calculated based on the Bray–Curtis dissimilarity matrices used “vegan” package (Wang et al., 2017). The “ggalluvial” package in was used to describe changes in bacteria and fungi (top 5 phyla with the highest abundance), and the “psych” package was used to calculate the correlation between top 5 phyla and soil properties, which was then visualized using the “ggplot2” package.

In order to better understand the connectivity and complexity of bacterial and fungal communities in *CF*, MF, and BF sample plots, we used “psych” package to calculate the Spearman correlation coefficients among OTUs in all samples, and the detection rate was greater than 77.7%. At the same time, the false discovery rate was used to correct the value of p multiplicity test. The rank correlation coefficient *r* > |0.9| and *p* < 0.001 were determined (Berry and Widder, 2014). Then, Cytoscape (3.7.1) was used for network visualization, and the number of OTUs at the phylum level was statistically analyzed. The top 6 modules with the highest OTUs enrichment in each network were selected for coloring. These modules were extracted by Cytoscape, colored with dominant phylum, and visualized using “ggplot2” package. The “psych” package was used to calculate the correlation between the OTU with the top 20 degrees in bacteria and fungi and soil properties, which was then visualized using the “ggplot2” package.

SPSS version 25.0 (SPSS Inc., Chicago, IL, United States) was used for one-way analysis of variance of soil physicochemical properties data, and Duncan’s multiple range test was used to identify the significant differences between means at a 5% significance level. All data are presented as the mean ± standard error (*n* = 9; Pan et al., 2021).

## Results

### Effects of stand differences on soil physicochemical properties and microbial alpha diversity

**Figure 2:**
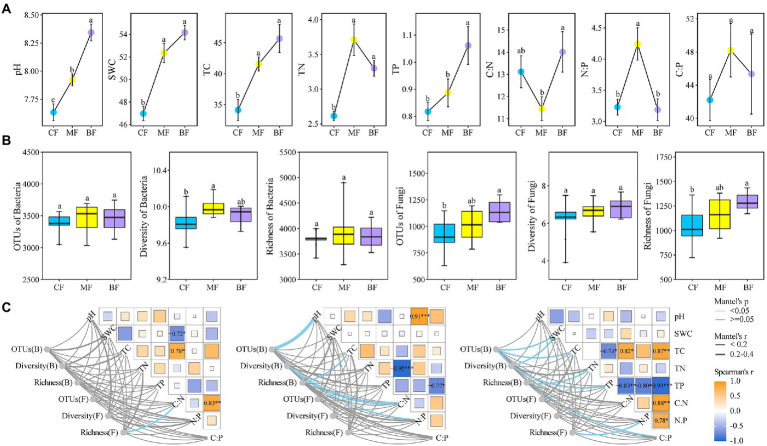
Soil physicochemical properties **(A)**, bacterial and fungal diversity **(B)** and their correlation **(C)** under forest stand differences. SWC, soil water content; TC, total carbon; TN, total nitrogen; TP, total phosphorus; OTUs, number of observed OTU. Diversity: Shannon index. Richness: ACE index. Values are mean ± standard errors (*n* = 9). Columns with different letters indicate significant differences at *p* < 0.05 (Duncan test). Pairwise comparisons of environmental factors are shown with a color gradient denoting Spearman’s correlation coefficient. *CF*, coniferous forest; MF, mixed forest; BF, broad-leaved forest.

With the succession from *CF* plot to BF plot, soil pH, SWC, TC and TP contents increased, and BF plot was significantly higher than *CF* plot (*p* < 0.05). It was worth noting that soil TN content in the MF plot was the highest and significantly higher than that in the *CF* plot. Whereas, soil C:N in the BF plot was higher than that in the *CF* and MF plots, respectively. In addition, soil N:P and C:P changed in the MF plot were similar to those in TN (Figure 2A).

Alpha diversity analysis showed that the number of bacterial OTUs and bacterial richness in the three sample plots were not significantly different, but the soil bacterial diversity index was higher in the MF sample plot and significantly higher than in the *CF* and BF sample plots. Interestingly, there was no significant difference in soil fungal community diversity among the three plots, while the number of fungal OTU and fungal richness were consistent, that is, with the succession from *CF* plot to BF plot, the number of fungal OTU and fungal richness increased, and BF plot was significantly higher than *CF* plot (*p* < 0.05; Figure 2B).

Further, the Mantel test showed that soil fungal richness in *CF* plot was significantly correlated with C:N (*p* < 0.05). The number of bacterial OTU was strongly correlated with pH, bacterial diversity was strongly correlated with C:N, and bacterial richness was strongly correlated with TC and C:P. With the succession from *CF* to BF plot, there was a correlation between the alpha diversity index of soil bacteria and fungi and the soil physicochemical properties (*p* < 0.05), such as bacterial OTUs and SWC, bacterial diversity and TP, bacterial richness and TN, and fungal richness and N:P index (Figure 2C).

### Effects of stand differences on assembly patterns of soil bacterial and fungal communities

**Figure 3:**
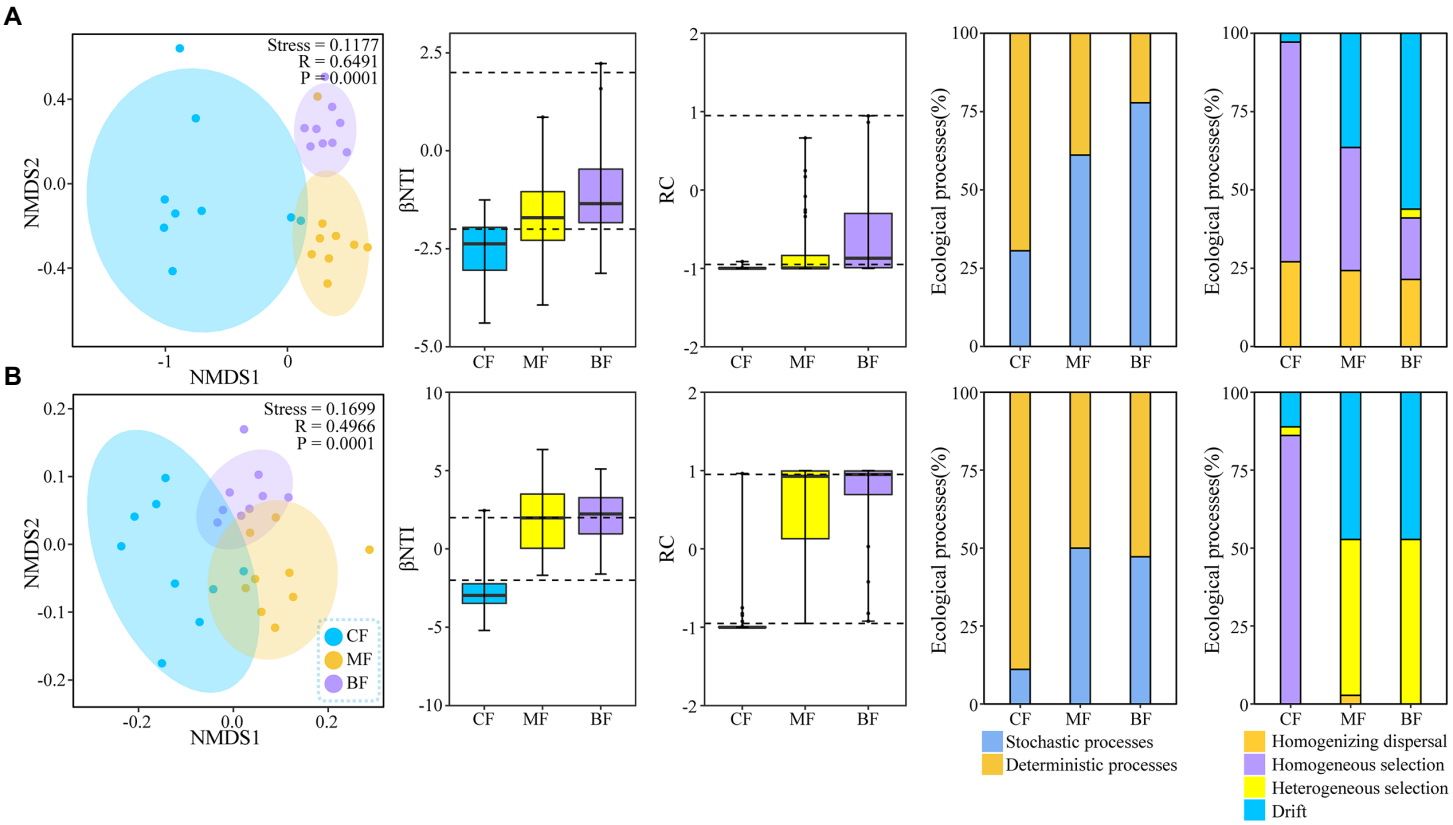
Non-metric multidimensional scaling ordination based on Bray-Curtis similarities and the ecological processes governing the compositional variation of soil bacterial **(A)** and fungal **(B)** communities under forest stand differences.

NMDS analysis showed that the composition of the soil bacterial (*R* = 0.6491, *p* = 0.0001) and fungal (*R* = 0.4966, *p* = 0.0001) communities were different among the three plots (Figure 3). NCM analysis showed that stochastic processes gradually dominated the soil bacterial community composition with succession from *CF* to BF plot (*R*^2^ = 0.785–0.831; Supplementary Figure S1). Further analysis by βNTI and null model showed that with the succession from *CF* to BF plot, the ecological processes of soil bacterial communities changed from deterministic processes (69.4% in *CF* plot) to stochastic processes (77.8% in BF plot). In *CF* plot, homogeneous selection (55.6%) was dominant; while in BF plot, drift (55.6%) was dominant. The ecological process of soil fungal community was similar to that of bacteria. However, homogeneous selection (86.1%) was dominant in *CF* plot, while heterogeneous selection (52.8%) and drift (47.2%) were dominant in BF plot (Figure 3).

**Figure 4:**
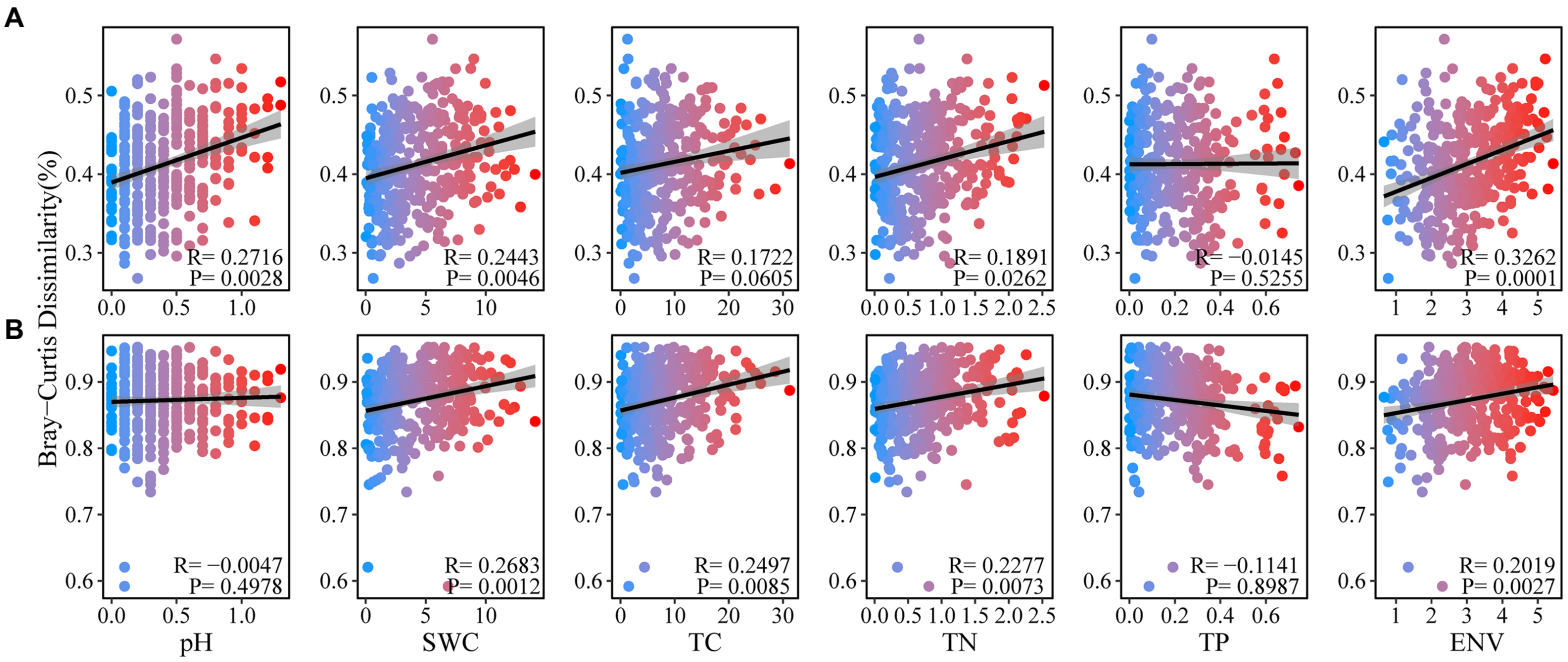
Distance–decay relationship for soil physicochemical properties with soil bacterial **(A)**, and fungal **(B)**, communities under forest stand differences.

In order to further explore the relationship between Bray–Curtis dissimilarity of soil bacteria and fungi and environmental factors, we found that Bray–Curtis dissimilarity of soil bacteria was positively correlated with soil pH, SWC and TN (Figure 4A). Fungal Bray-Curits dissimilarity was significantly positively correlated with soil SWC, TC and TN (*p* < 0.05; Figure 4B). The Bray–Curtis dissimilarity of soil bacteria and fungi were significantly correlated with environmental factors (*p* < 0.05).

### Effects of stand differences on soil bacterial and fungal community structure

**Figure 5:**
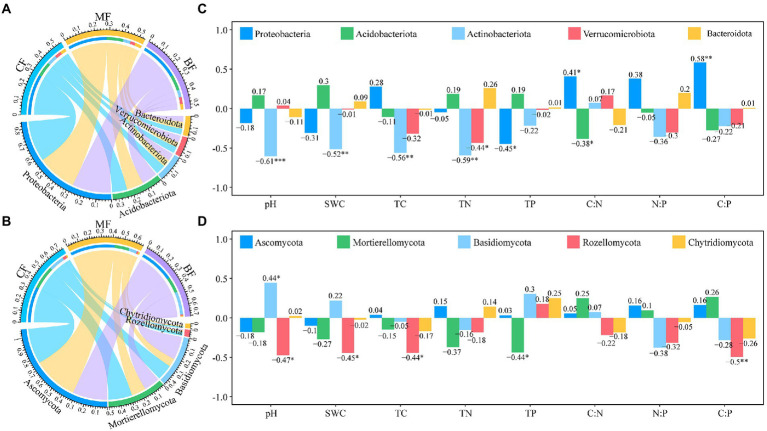
Microbial composition of bacteria **(A)**, and fungi **(C)**, at the phylum level, and the correlation between soil physicochemical properties and top 5 phylum in the bacterial **(B)**, and fungal **(D)**, communities under forest stand differences.

Of the 27 soil samples, 84.57% OTUs of bacteria and 36.40% OTUs of fungi were annotated at the phylum level. With the succession from *CF* to BL plot, the relative abundance of Proteobacteria (29.2–27.6%), Actinobacteriota (7.1–5.3%) and Bacteroidota (4.1–4.0%) decreased, while the relative abundance of Acidobacteriota (10.7–11.2%) and Verrucomicrobiota (4.6–5.0%) increased (Figure 5A). The relative abundance of Ascomycota (33.1–32.3%), Mortierellomycota (22.2–14.9%), Rozellomycota (3.6–1.1%) and Chytridiomycota (1.3–1.1%) decreased, while the relative abundance of Basidiomycota (14.8–21.2%) increased (Figure 5B).

The correlation between the relative abundance of dominant phyla and the soil physicochemical properties was further analyzed. Actinobacteriota were significantly negatively correlated with soil pH, SWC, TC and TN (*p* < 0.05), while Proteobacteria and Verumobacteria were significantly negatively correlated with TP and TN, respectively (Figure 5C). Rozellomycota was significantly negatively correlated with soil pH, SWC and TC, while Mortieromycota was significantly negatively correlated with TP (*p* < 0.05; Figure 5D).

The top 20 bacterial genera in relative abundance of three sample plots were *MND1*, *Candidatus Udaeobacter*, *Sphingomonas*, *RB41*, *Brevundimonas*, *Haliangium*, *Escherichia Shigella*, *Bradyrhizobium*, *IS44*, *Rhodoplanes*, *Ellin6055*, *Candidatus Solibacter*, *Ellin6067*, *Dongia*, *Bryobacter*, *Agathobacter*, *Gaiella*, *Terrimonas*, *Pedomicrobium* and *Subgroup 10* (Supplementary Figure S2A). And *MND1*, *RB41*, *Candidatus Solibacter*, *Ellin6067*, and *Subgroup 10* were the differential genera of top 20 bacterial genera in relative abundance in *CF* and BF sample plots (Supplementary Figure S2B). While the top 20 fungal genera in relative abundance of three sample plots were *Mortierella*, *Fusarium*, *Inocybe*, *Tuber*, *Pseudogymnoascus*, *Penicillium*, *Cladosporium*, *Hymenogaster*, *Malassezia*, *Pichia*, *Trichophaea*, *Trichocladium*, *Aspergillus*, *Solicoccozyma*, *Chaetomium*, *Protoglossum*, *Archaeorhizomyces*, *Phallus*, *Geminibasidium* and *Dactylonectria* (Supplementary Figure S3A). However, there were no differential genera of top 20 fungal genera in relative abundance in *CF* and BF sample plots (Supplementary Figure S3B).

### Effects of stand differences on the structure and co-occurrence network of soil bacterial and fungal communities

**Figure 6:**
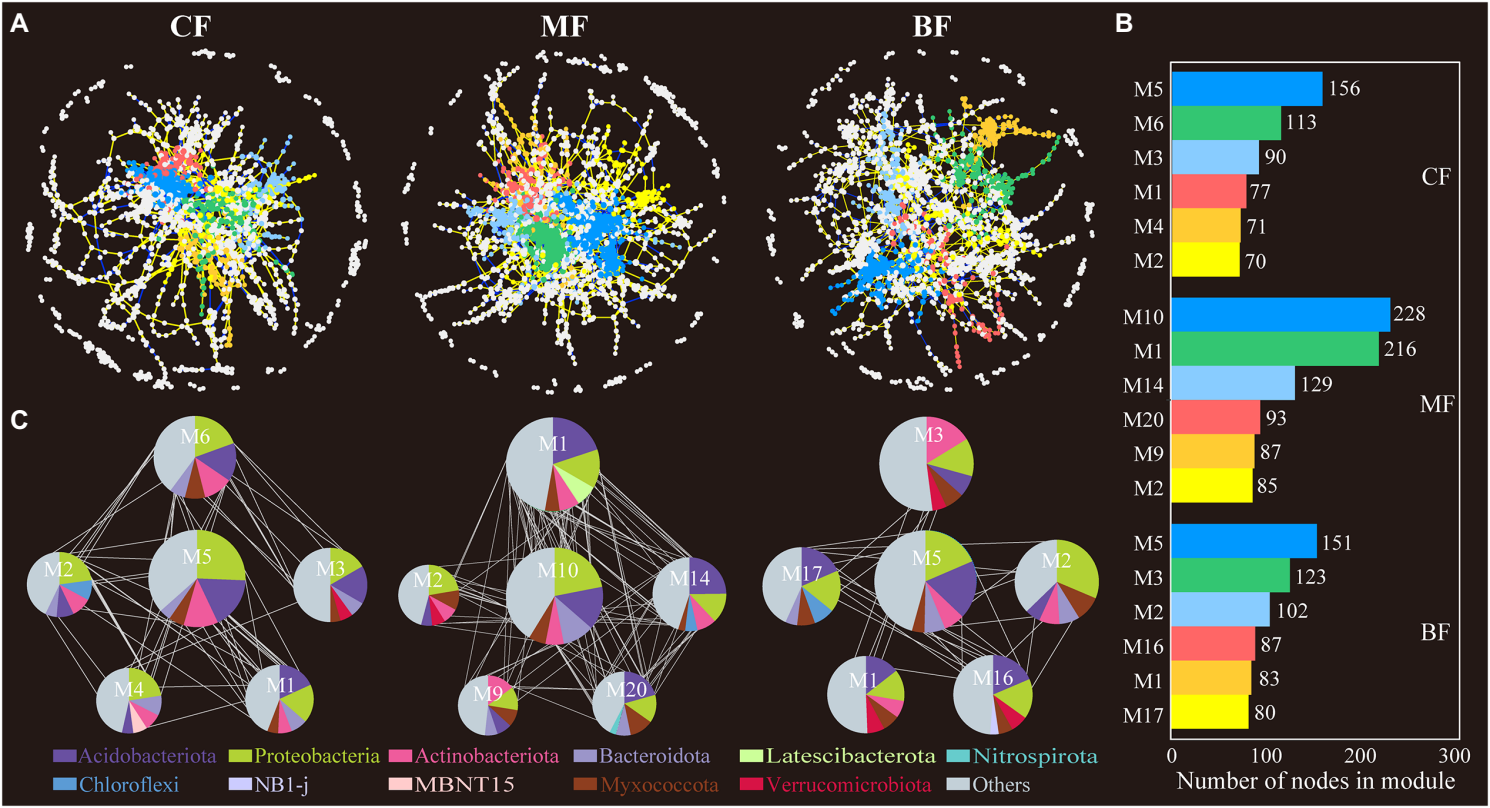
Bacterial co-occurrence network **(A)** and the module analysis **(B,C)** based on correlation analysis under forest stand differences. The size of each node represents the degree. The yellow lines indicate positive interactions, blue lines indicate negative interactions, while the gray lines indicate correlations between each module.

The co-occurrence network showed that the network connectivity and complexity differed between soil bacteria and fungi. In the bacterial network, the network relationship in the MF plot was more complex (1,818 nodes and 3,516 edges), and the BF plot (1,798 nodes and 2,831 edges) had higher network connectivity and complexity than the *CF* plot (1,624 nodes and 2,676 edges). The number of soil bacterial network modules in *CF* plot (105) was also higher than that in MF (87) and BF (88) plots. In addition, the positive edges, negative edges and average degree of soil bacterial network in MF (2,573 positive edges, 943 negative edges) were higher than those in *CF* (1,950 positive edges, 729 negative edges) and BF (2,122 positive edges, 709 negative edges) plots. It was worth noting that the BF plot had the highest positive/negative edge (2.99). The average path distance of BF plot (10.67) was higher than that of *CF* (10.24) and MF (8.79), and the harmonic geodesic distance of BF plot (9.26) was also higher than that of *CF* (8.46) and MF (7.61; Figure 6; Supplementary Table S1).

**Figure 7:**
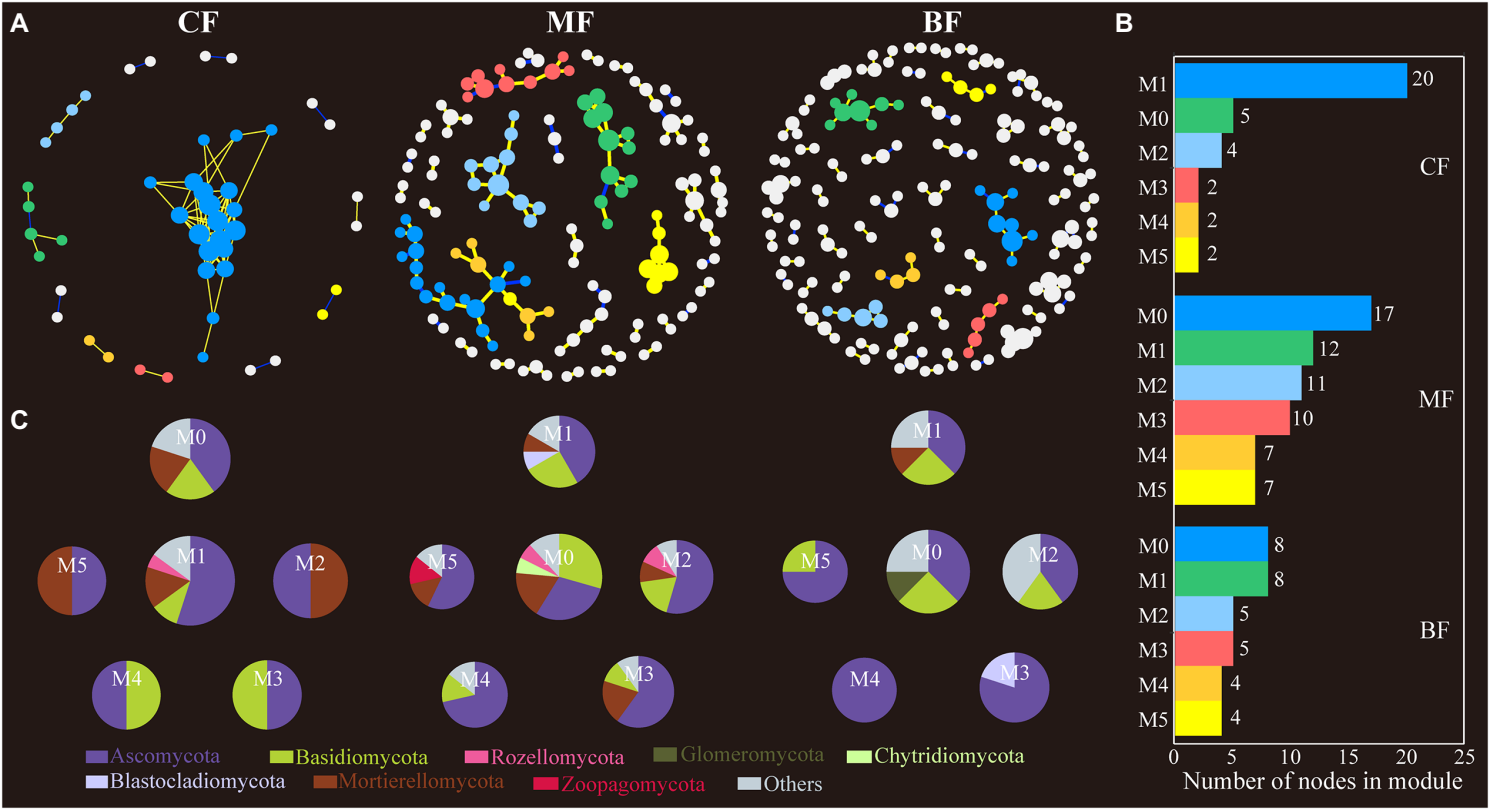
Fungal co-occurrence network **(A)** and the module analysis **(B,C)** based on correlation analysis under forest stand differences. The size of each node represents the degree. The yellow lines indicate positive interactions, blue lines indicate negative interactions, while the gray lines indicate correlations between each module.

In the soil fungal network, the connectivity and complexity of the network gradually increased with the succession of *CF* plot to BF plot (the number of nodes from 47 to 149, and the number of edges from 100 to 125). The number of soil fungal network modules in BF (65) plot was also higher than that in *CF* (37) and MF (12) plots (Figure 7; Supplementary Table S1).

**Figure 8:**
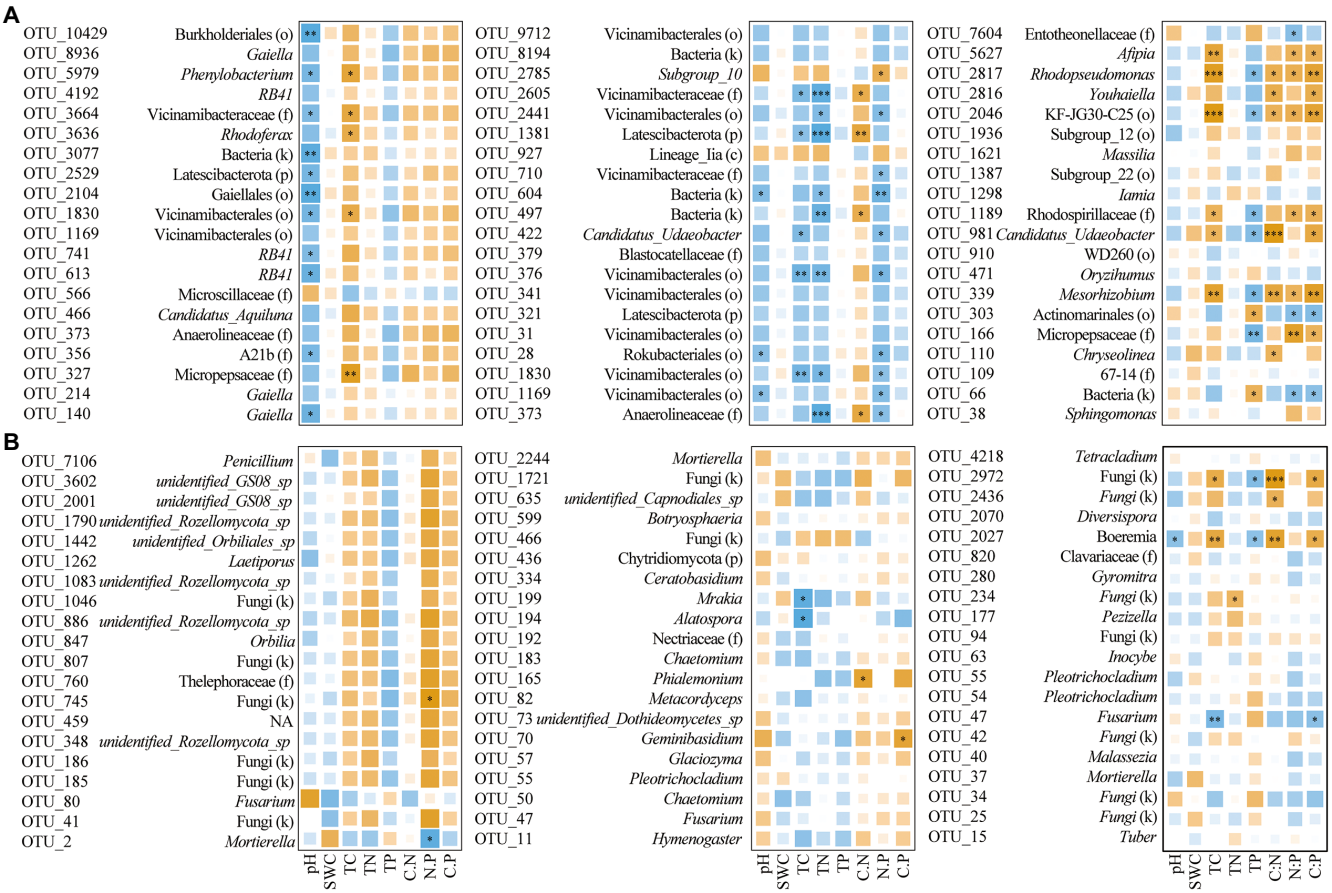
Correlation between OTU with the top 20° in co-occurrence networks of bacteria **(A)** and fungi **(B)** with soil physicochemical properties under forest stand differences (^*^p < 0.05; ^**^p < 0.01; ^***^p < 0.001).

In the network analysis, the OTUs in the main modules of soil bacteria in the three sample plots were dominated in Acidobacteriota, Proteobacteria, Actinobacteriota, and Bacteroidota; in fungi, Ascomycota, Basidiomycota and Mortierellomycota were predominant. The top 20 OTUs of each network in terms of degree were selected, and correlation analysis with soil physicochemical properties showed that the magnitude of the bacterial network degree was closely related to physicochemical properties. Among them, soil bacteria in *CF* showed a negative correlation with pH and a positive correlation with TC; soil bacteria in MF showed a negative correlation with TC, TN, and C:P ratio; and in BF, soil bacteria showed a positive correlation with TC, C:N, N:P and C:P ratio (*p* < 0.05; Supplementary Figure S3; Figure 8).

## Discussion

### Stand differences changed soil physicochemical properties and microbial community diversity

In this study, the results showed significant soil pH, SWC, TC, TN, and TP differences among *CF*, MF, and BF (*p* < 0.05; Figure 2A). Differences in plant species have been reported to lead to changes in soil properties caused by species differences affecting root exudates and litter, resulting in an unbalanced input of soil nutrients (Li C. Y. et al., 2021). In general, the litter of BF contributes significantly to soil C, N, and P accumulation (Pang et al., 2020). In this study, the soil TC, TN, and TP contents in BF plot were higher than those of MF and *CF.* Previous study pointed out that the difference in litter quality, the leaves of broad-leaved forest were larger and decomposed faster than those of coniferous forests, which was conducive to the accumulation of nutrients in the soil, whereas coniferous forests were the opposite (Mueller et al., 2015). Soil pH and TC content increased in the succession from coniferous forest to broad-leaved forest (Qu et al., 2020), and similar conclusions were obtained in this study. Interestingly, the MF maintained high N:P ratio. The existing studies suggested that the leaves of trees, shrubs and herbaceous plants in mixed forests maintain high N:P ratios (Pang et al., 2020). With the input of plant leaf litter to soil nutrients, the reason why N:P was higher in MF plot than *CF* and BF in this study was speculated. It was noteworthy that the N:P ratios of the three sample plots were all less than 10, indicating that soil in Liupanshan Mountain was obviously limited by nitrogen (Elisabeth and Brent, 2013).

Vegetation type can indirectly change the diversity and richness of soil bacterial and fungal communities by changing the soil physicochemical properties (Yao et al., 2017; Deng et al., 2019). In this study, there were differences in soil bacterial community diversity among the three sample plots (Figure 2B). A recent study indicated that the coniferous and broad-leaved mixed forest had higher soil bacterial alpha diversity, which was due to the higher soil TN (Li et al., 2014). In addition, the richness of soil fungal community increased with the succession from *CF* to BF plot (Figure 2B). It has been reported that coniferous and broad-leaved tree species mixing can increase the richness of soil fungal community and subsequently affect the structure of soil fungal community, which further demonstrated that differences in soil physicochemical properties (temperature, soil moisture, litter, TN, C:N and N:P ratio) were important factors affecting the structure of soil microbial community (Dong et al., 2021).

According to Mantel test, soil fungal community richness was significantly correlated with soil C:N in *CF* plot (*p* < 0.05; Figure 2C). Because coniferous forest had more difficult to degrade lignin and coarse fiber, these substances were difficult to be broken down by microorganisms. Since soil fungi participate in the decomposition of recalcitran organic matter (lignin), fungi that were more dependent on soil nutrients have a strong correlation with C:N (de Boer et al., 2005; Šnajdr et al., 2008). This is consistent with earlier findings and provides new evidence revealing the effects of tree species differences on soil properties and microbial communities in temperate forests (Gao et al., 2019; Sheng et al., 2019). An increasing number of studies have recorded the response of soil microbial community diversity to environmental changes (de Carvalho et al., 2016; Tripathi et al., 2016). Among them, soil bacterial diversity and community composition were observed to be strongly correlated with soil pH (Rousk et al., 2010).

### Stochastic ecological processes of the soil microbial community dominated during the succession from *CF* to MF

Deterministic and stochastic ecological processes represent complementary and continuous ecological processes of assembly patterns in microbial communities (Zhou and Ning, 2017). In our study, the driving effect of stochastic processes on soil bacterial and fungal communities gradually increased as the sample plots changed from *CF* to MF (Supplement Figure S1). Therefore, we infer that there is a unified continuum between deterministic and stochastic processes in the microbial community in our study area, which is consistent with the results of previous studies (Chase et al., 2011; Tian et al., 2017). Myers et al. (2013) indicated that the assembly patterns of soil microbial communities in temperate forests reflected stronger environmental correlations, whereas plant species differences played a key role. With succession from *CF* to BF, the stochastic process of the soil bacterial community gradually dominated. The study further showed that the process of bacterial community assembly patterns also changes with the long-term change of vegetation cover (Goss-Souza et al., 2017). In the early stages of succession, the assembly patterns of soil bacterial communities were dominated by stochastic processes, whereas in the late stages of succession, deterministic processes gradually dominated (Ferrenberg et al., 2013; Dini-Andreote et al., 2015). In this study, *L. principis-rupprechtii* was planted early and for a long time, confirming the above conclusions. In addition, the succession from *CF* to BF was driven but not dominated by stochastic processes in the soil fungal community (Figure 3). One explanation for the differences in the assembly patterns of bacterial and fungal communities is that fungal communities are initially strongly dominated by deterministic processes; however, this dominance gradually diminishes as succession proceeds along the temporal timescale (Tian et al., 2017). Nonetheless, soil bacterial communities have more remarkable metabolic functional plasticity (Massana and Logares, 2013), which may be because the deviation level of soil fungal communities is lower than that of soil bacterial communities. This also explains our first hypothesis that stochastic and deterministic processes influence community assembly patterns of soil bacteria and fungi in temperate forests.

Environmental heterogeneity was the main factor affecting the geographical distribution of the microbial communities (Zhou et al., 2013; Liu et al., 2019). For example, soil salinity, pH, carbon and nitrogen content, and the C:N ratio may all be determinants of the soil microbial community (Griffiths et al., 2011; Xiong et al., 2014). In this study, the soil bacterial and fungal communities were affected by environmental factors. Soil pH and SWC content were strongly correlated with the assembly patterns of the soil bacterial communities (Figure 4). Previous studies have shown that the degree to which stochastic and deterministic ecological processes in the soil bacterial community are affected by soil pH (Tripathi et al., 2018), and SWC also explains the changes in soil bacterial communities (Delgado-Baquerizo et al., 2018). Soil SWC and TN content were significantly correlated with fungal communities (*p* < 0.05), and previous studies have shown that changes in bacterial communities largely depended on changes in soil pH (Rousk et al., 2010). In contrast, the main influences on fungal communities tended to be soil carbon, moisture, and plant species and were not sensitive to changes in soil pH (Barberán et al., 2015; Prober et al., 2015). In our study, the soil TN content of *CF* was significantly different from that of MF and BF, which highlighted the critical role of soil physicochemical properties in fungal community assembly.

### Soil bacterial and fungal communities in MF had more complex network

Soil bacterial and fungal communities with different ecological functions play important roles in forest ecosystems. In our study, Actinobacteriota had a strong correlation with pH, SWC, TC and TN (Figure 5). It has been shown that Actinobacteriota, as k-strategists oligotrophic bacteria, depends on the availability of water and nutrients (Li et al., 2022), and can play an important role in microbial co-occurrence networks (Chen et al., 2021). Our study further confirmed the above conclusions. In addition, a study on forest fungal diversity concluded that changes in total soil carbon and nitrogen content during forest succession were negatively correlated with the relative abundance of Rozellomycota (Bai et al., 2019), which explained the decrease in the relative abundance of Rozellomycota during the succession from *CF* to BF sample plots in this study and the significant correlation with soil TC and C:P. It has been hypothesized that an increase in the relative abundance of Mortierellomycota decreases soil microbial diversity (Li X. et al., 2021), and our study found that the relative abundance of Mortierellomycota decreased with the succession from *CF* to BF sample plots and was significantly correlated with soil TP content (*p* < 0.05). These results further proved that the change of soil available nutrients during forest succession was determined by the interaction between microorganisms and litter inputs of different quality.

Network analysis to study microbial co-occurrence patterns can better reveal interactions between ecological niches and microbial taxa (Barberán et al., 2012). We constructed bacterial and fungal co-occurrence networks for each of the three sample plots and found that the microbial co-occurrence networks did not occur randomly. However, communities or OTUs formed modular groupings of differently associated species (Barberán et al., 2012; Sul et al., 2013). In network analysis, a higher degree of modularity and denser network recorded a highly complex microbial community (Olesen et al., 2007). In this study, the number of soil bacterial network modules was the largest in *CF*, and the number of soil fungal network modules was the largest in BF (Figures 6, 7), which has been confirmed in studies related to soil bacterial and fungal community co-occurrence networks during forest ecosystem restoration (Sun et al., 2017); that is, the bacterial co-occurrence networks became more complex as succession proceeded (Avera et al., 2015; Xue et al., 2017). In addition, the number of nodes and edges in the network further characterizes the connectivity of the network. The nodes and edges of soil bacteria in *CF* were lower than those in MF and BF, and the positive/negative edge ratios were the lowest in this study (Supplementary Table S1). This implies that in the co-occurrence network, microorganisms compete for ecological niches favorable to their growth needs through competition and complementarity (Faust and Raes, 2012). During grassland restoration, microorganisms avoid grazing intrusion through ecological niche competition to maintain the soil ecosystem function (Yao et al., 2018; Cong et al., 2021). In another study, with seasonal changes, plant rhizosphere bacterial communities responded to utilizing and acquiring soil nutrients during the plant growth stages through a complementary ecological niche strategy (Shi et al., 2016). The average path distance in the network highlighted the shortest path between two nodes. From an ecological point of view, the number of modules and complexity of paths characterizes the cluster species as performing more functions and roles in the network (Wang et al., 2016). In this study, the fungal communities in the *CF* differed from those in the MF and BF and had more complex network relationships, indicating that the fungal communities in the *CF* were more competitive. In general, abundant resources favored taxa selection and led to enhanced interactions among OTUs in the network, whereas soil nutrient limitation intensified resource grabbing among taxa and formed closer interrelationships (Pan et al., 2021).

We further analyzed the correlation between the top 20° of the major taxa in each network and the physicochemical properties, and found that with the succession from *CF* to BF plots, the correlation between the major taxa in each network and the physicochemical properties of soil was different. For example, bacterial taxa in the *CF* plot were almost negatively correlated with pH, but strongly positively correlated with TC (*p* < 0.05; Figure 8). Members such as *Gaiella* and *RB41* are known to be associated with inputs to soil pH and carbon inputs (Lin et al., 2019; Wang et al., 2022). This further confirmed the influence of refractory carbon input in coniferous forests on soil microbial interactions. In the MF plot, members of Vicinamibacterales were mostly negatively correlated with soil TC and TN. In view of the potential role of Vicinamibacterales in biological antagonism and ecological restoration (Chun et al., 2021; Xu et al., 2022); it is speculated that they can be used as indicator taxa of mixed temperate forest and regulated by soil carbon and nitrogen. Major taxa such as Afipia, *Rhodopseudomonas* and *Mesorhizobium* actively participated in the soil carbon, nitrogen and phosphorus cycling, and the significant correlation with physicochemical properties in the BF plot further validated the results of previous studies (LaSarre et al., 2017; Duan et al., 2020; Luo et al., 2020). In addition, the dependence of soil fungal communities on carbon resources in BF plot further indicated that the interactions of major taxa could further stabilize the microbial community structure. This also confirmed our second hypothesis that succession from *CF* to MF and BF plots changed the network relationship of soil bacterial and fungal communities.

## Conclusion

The distribution of soil microorganisms is heterogeneous even within the same land-use type, and the heterogeneity of the soil environment primarily causes this variation. During the succession from coniferous to broad-leaved forests in temperate forests, the soil SWC, TC, TN and TP contents of broad-leaved forests increased, as did the number and richness of soil fungal communities. In the succession process from *CF* to BF plot, the composition of soil bacterial and fungal communities changed from deterministic to stochastic process, and the differences of soil bacterial and fungal communities were strongly correlated with environmental factors. In addition, the succession from coniferous to broad-leaved forests in temperate forests is beneficial for maintaining the complex network relationships of soil bacteria and fungi, and the major taxa in the soil bacterial network in broad-leaved forests was strongly correlated with soil carbon, nitrogen and phosphorus cycling. Our study provides a theoretical basis for soil microbial interactions under temperate forest stand differences, not only showing the mechanism by which species differences in temperate forests of northern China affect soil microbial community assembly patterns but also emphasizing the importance of the soil microbiome as a key factor in ecosystems through co-occurrence networks.

## Data availability statement

The datasets presented in this study can be found in online repositories. The names of the repository/repositories and accession number(s) can be found at: https://www.ncbi.nlm.nih.gov/, PRJNA867578, PRJNA868111.

## Author contributions

PK and YP conceived and designed the study. YP, PY, JH, TZ, and YZ did running the experiments and data management. JH, YP, and XD performed the data mining, statistical analysis, interpretation, and figure preparation of sequencing results. PK, YP, and XY did the manuscript writing and revising. PK and YP contributed equally. All authors contributed to the article and approved the submitted manuscript.

## Funding

This work was supported financially by Natural Science Foundation of Ningxia Province (2022AAC03227), the Key Research and Development Program of Ningxia (2018BEG02001, 2019BEB04018), and Innovation Team for Genetic Improvement of Economic Forests Foundation (2022QCXTD04).

## Conflict of interest

The authors declare that the research was conducted in the absence of any commercial or financial relationships that could be construed as a potential conflict of interest.

## Publisher’s note

All claims expressed in this article are solely those of the authors and do not necessarily represent those of their affiliated organizations, or those of the publisher, the editors and the reviewers. Any product that may be evaluated in this article, or claim that may be made by its manufacturer, is not guaranteed or endorsed by the publisher.

## Footnotes

^1^ https://ccb.jhu.edu/software/FLASH/index.shtmsl

^2^ https://github.com/Hy4m/linkET

## References

[ref1] Avera B. N. Strahm B. D. Burger J. A. Zipper C. E. (2015). Development of ecosystem structure and function on reforested surface-mined lands in the central Appalachian Coal Basin of the United States. New For. 46, 683–702. doi: 10.1007/s11056-015-9502-8

[ref2] Bai Z. Wu X. Lin J. J. Xie H. T. Yuan H. S. Liang C. (2019). Litter-, soil- and C:N-stoichiometry-associated shifts in fungal communities along a subtropical forest succession. Catena 178, 350–358. doi: 10.1016/j.catena.2019.03.037

[ref3] Bao S. D. (2000). Soil and Agricultural Chemistry Analysis. China Agriculture Publication.

[ref4] Barberán A. Bates S. T. Casamayor E. O. Fierer N. (2012). Using network analysis to explore co-occurrence patterns in soil microbial communities. ISME J. 6, 343–351. doi: 10.1038/ismej.2011.119, PMID: 21900968 PMC3260507

[ref5] Barberán A. McGuire K. L. Wolf J. A. Jones F. A. Wright S. J. Turner B. L.. (2015). Relating belowground microbial composition to the taxonomic, phylogenetic, and functional trait distributions of trees in a tropical forest. Ecol. Lett. 18, 1397–1405. doi: 10.1111/ele.12536, PMID: 26472095

[ref6] Berry D. Widder S. (2014). Deciphering microbial interactions and detecting keystone species with co-occurrence networks. Front. Microbiol. 5:219. doi: 10.3389/fmicb.2014.00219, PMID: 24904535 PMC4033041

[ref7] Bettinelli M. Beone G. M. Spezia S. Baffi C. (2000). Determination of heavy metals in soils and sediments by microwave-assisted digestion and inductively coupled plasma optical emission spectrometry analysis. Anal. Chim. Acta 424, 289–296. doi: 10.1016/S0003-2670(00)01123-5

[ref8] Bi B. Y. Zhang H. Yuan Y. Wu Z. H. Wang Y. Han F. (2021). Dynamic changes of soil microbial community in *Pinus sylvestris var. mongolica* plantations in the Mu us Sandy land. J. Environ. Manag. 287:112306. doi: 10.1016/j.jenvman.2021.112306, PMID: 33714736

[ref9] Caporaso J. G. Lauber C. L. Walters W. A. Berg-Lyons D. Huntley J. Fierer N.. (2012). Ultra-high-throughput microbial community analysis on the illumina HiSeq and MiSeq platforms. ISME J. 6, 1621–1624. doi: 10.1038/ismej.2012.8, PMID: 22402401 PMC3400413

[ref10] Chang Y. Y. Chen F. Zhu Y. F. You Y. N. Cheng Y. J. Ma J. (2022). Influence of revegetation on soil microbial community and its assembly process in the open-pit mining area of the loess plateau, China. Front. Microbiol. 13:992816. doi: 10.3389/fmicb.2022.992816, PMID: 36090080 PMC9453671

[ref11] Chase J. M. Kraft N. J. B. Smith K. G. Vellend M. Inouye B. D. (2011). Using null models to disentangle variation in community dissimilarity from variation in α-diversity. Ecosphere 2:art24. doi: 10.1890/ES10-00117.1

[ref12] Chen L. F. He Z. B. Wu X. R. Du J. Zhu X. Lin P. F.. (2021). Linkages between soil respiration and microbial communities following afforestation of alpine grasslands in the northeastern Tibetan plateau. Appl. Soil Ecol. 161:103882. doi: 10.1016/j.apsoil.2021.103882

[ref13] Chen W. D. Ren K. X. Isabwe A. Chen H. H. Liu M. Yang J. (2019). Stochastic processes shape microeukaryotic community assembly in a subtropical river across wet and dry seasons. Microbiome 7:138. doi: 10.1186/s40168-019-0749-8, PMID: 31640783 PMC6806580

[ref14] Chen J. Wang P. F. Wang C. T. Wang X. Miao L. Z. Liu S.. (2019). Fungal community demonstrates stronger dispersal limitation and less network connectivity than bacterial community in sediments along a large river. Environ. Microbiol. 22, 832–849. doi: 10.1111/1462-2920.14795, PMID: 31469494

[ref15] Chun S. J. Kim Y. J. Cui Y. Nam K. H. (2021). Ecological network analysis reveals distinctive microbial modules associated with heavy metal contamination of abandoned mine soils in Korea. Environ. Pollut. 289:117851. doi: 10.1016/j.envpol.2021.117851, PMID: 34358869

[ref16] Cong W. Yu J. J. Feng K. Deng Y. Zhang Y. G. (2021). The coexistence relationship between plants and soil bacteria based on interdomain ecological network analysis. Front. Microbiol. 12:745582. doi: 10.3389/fmicb.2021.745582, PMID: 34950114 PMC8689066

[ref17] de Boer W. Larissa B. F. Richard C. S. Lynne B. (2005). Living in a fungal world: impact of fungi on soil bacterial niche development. FEMS Microbiol. Rev. 29, 795–811. doi: 10.1016/j.femsre.2004.11.005, PMID: 16102603

[ref18] de Carvalho T. S. Jesus E. D. Barlow J. Gardner T. A. Soares I. C. Tiedje J. M.. (2016). Land use intensification in the humid tropics increased both alpha and beta diversity of soil bacteria. Ecology 97, 2760–2771. doi: 10.1002/ecy.1513, PMID: 27859123

[ref19] Delgado-Baquerizo M. Oliverio A. M. Brewer T. E. Benavent-González A. Eldridge D. J. Bardgett R. D.. (2018). A global atlas of the dominant bacteria found in soil. Science 359, 320–325. doi: 10.1126/science.aap9516, PMID: 29348236

[ref20] Deng J. Yin Y. Luo J. Zhu W. Zhou Y. (2019). Different revegetation types alter soil physical-chemical characteristics and fungal community in the Baishilazi nature reserve. Peer J. 6:e6251. doi: 10.7717/peerj.6251, PMID: 30648009 PMC6330947

[ref21] Dini-Andreote F. Stegen J. C. van Elsas J. D. Salles J. F. (2015). Disentangling mechanisms that mediate the balance between stochastic and deterministic processes in microbial succession. Proc. Natl. Acad. Sci. U. S. A. 112, E1326–E1332. doi: 10.1073/pnas.1414261112, PMID: 25733885 PMC4371938

[ref22] Dong X. D. Gao P. Zhou R. Li C. Dun X. J. Niu X. (2021). Changing characteristics and influencing factors of the soil microbial community during litter decomposition in a mixed *Quercus acutissima* Carruth. And *Robinia pseudoacacia* L. forest in northern China. Catena 196:104811. doi: 10.1016/j.catena.2020.104811

[ref23] Duan C. W. Liu Y. Zhang H. G. Chen G. Y. Song J. F. (2020). Cadmium pollution impact on the bacterial community of haplic cambisols in Northeast China and inference of resistant genera. J. Soil Sci. Plant Nutr. 20, 1156–1170. doi: 10.1007/s42729-020-00201-5

[ref24] Elisabeth N. B. Brent L. H. (2013). C:N:P stoichiometry in Australian soils with respect to vegetation and environmental factors. Plant Soil 373, 553–568. doi: 10.1007/s11104-013-1823-9

[ref25] Faust K. Raes J. (2012). Microbial interactions: from networks to models. Nat. Rev. Microbiol. 10, 538–550. doi: 10.1038/nrmicro2832 22796884

[ref26] Feng Y. Chen R. Stegen J. C. Guo Z. Zhang J. Li Z.. (2018). Two key features influencing community assembly processes at regional scale: initial state and degree of change in environmental conditions. Mol. Ecol. 27, 5238–5251. doi: 10.1111/mec.14914, PMID: 30368967

[ref27] Ferrenberg S. O’Neill S. P. Knelman J. E. Todd B. Duggan S. Bradley D.. (2013). Changes in assembly processes in soil bacterial communities following a wildfire disturbance. ISME J. 7, 1102–1111. doi: 10.1038/ismej.2013.11, PMID: 23407312 PMC3660671

[ref28] Gao Y. Zhang X. He G. Shchepin O. N. Yan S. Chen S. (2019). Influence of forest type on dark-spored myxomycete community in subtropical forest soil, China. Soil Biol. Biochem. 138:107606. doi: 10.1016/j.soilbio.2019.107606

[ref29] Goss-Souza D. Mendes L. W. Borges C. D. Baretta D. Tsai S. M. Rodrigues J. L. M. (2017). Soil microbial community dynamics and assembly under long-term land use change. FEMS Microbiol. Ecol. 93:fix109. doi: 10.1093/femsec/fix109, PMID: 28961809

[ref30] Griffiths R. I. Thomson B. C. James P. Bell T. Bailey M. Whiteley A. S. (2011). The bacterial biogeography of British soils. Environ. Microbiol. 13, 1642–1654. doi: 10.1111/j.1462-2920.2011.02480.x, PMID: 21507180

[ref31] Haas B. J. Gevers D. Earl A. M. Feldgarden M. Ward D. V. Giannoukos G.. (2011). Chimeric 16S rRNA sequence formation and detection in sanger and 454-pyrosequenced PCR amplicons. Genome Res. 21, 494–504. doi: 10.1101/gr.112730.110, PMID: 21212162 PMC3044863

[ref32] Jiao S. Chu H. Zhang B. Wei X. Chen W. Wei G. (2022). Linking soil fungi to bacterial community assembly in arid ecosystems. iMeta. 1:e2. doi: 10.1002/imt2.2 PMC10989902 38867731

[ref33] Kane J. L. Morrissey E. M. Skousen J. G. Freedman Z. B. (2020). Soil microbial succession following surface mining is governed primarily by deterministic factors. FEMS Microbiol. Ecol. 96:fiaa114. doi: 10.1093/femsec/fiaa114, PMID: 32510564

[ref34] Kang L. Y. Chen L. Y. Zhang D. Y. Peng Y. F. Song Y. T. Kou D.. (2022). Stochastic processes regulate belowground community assembly in alpine grasslands on the Tibetan plateau. Environ. Microbiol. 24, 179–194. doi: 10.1111/1462-2920.15827, PMID: 34750948

[ref35] Kuczynski J. Stombaugh J. Walters W. A. Gonzalez A. Caporaso J. G. Knight R. (2012). Using QIIME to analyze 16S rRNA gene sequences from microbial communities. Curr. Protoc. Microbiol. Chapter 1:Unit 1E.5. doi: 10.1002/9780471729259.mc01e05s27, PMID: 23184592 PMC4477843

[ref36] LaSarre B. McCully A. L. Lennon J. T. McKinlay J. B. (2017). Microbial mutualism dynamics governed by dose-dependent toxicity of cross-fed nutrients. ISME J. 11, 337–348. doi: 10.1038/ismej.2016.141, PMID: 27898053 PMC5270580

[ref38] Li C. Y. Li X. L. Su X. X. Yang Y. W. Li H. L. (2021). Effects of alpine wetland degradation on soil microbial structure and diversity on the Qinghai Tibet plateau. Eurasian Soil Sci. 54, S33–S41. doi: 10.1134/S1064229322030097

[ref39] Li X. Liu X. Xie J. Zhang Q. Yang Z. Schindlbacher A.. (2021). Contribution of above ground litterfall and roots to the soil CO_2_ efflux of two sub-tropical *Cunninghamia lanceolata* and *Castanopsis carlesii* forests. Agric. For. Meteoro. 311:108671. doi: 10.1016/j.agrformet.2021.108671

[ref40] Li H. Ye D. D. Wang X. G. Settles M. L. Wang J. Hao Z.. (2014). Soil bacterial communities of different natural forest types in Northeast China. Plant Soil 383, 203–216. doi: 10.1007/s11104-014-2165-y

[ref41] Li M. Zhang K. R. Yan Z. Q. Liu L. Kang E. Kang X. M. (2022). Soil water content shapes microbial community along gradients of wetland degradation on the Tibetan plateau. Front. Microbiol. 13:824267. doi: 10.3389/fmicb.2022.824267, PMID: 35185848 PMC8847787

[ref42] Lian F. Xing B. S. (2017). Black carbon (biochar) in water/soil environments: molecular structure, sorption, stability, and potential risk. Environ. Sci. Technol. 51, 13517–13532. doi: 10.1021/acs.est.7b02528, PMID: 29116778

[ref43] Lin Y. X. Ye G. P. Kuzyakov Y. Liu D. Y. Fan J. B. Ding W. X. (2019). Long-term manure application increases soil organic matter and aggregation, and alters microbial community structure and keystone taxa. Soil Biol. Biochem. 134, 187–196. doi: 10.1016/j.soilbio.2019.03.030

[ref300] Lin Y. T. Whitman W. B. Coleman D. C. Jien S. H. Wang H. C. Chiu C. Y. (2021). Soil bacterial communities at the treeline in subtropical alpine areas. Catena 201:105205. doi: 10.1016/j.catena.2021.105205

[ref44] Liu M. Sui X. Hu Y. Feng F. (2019). Microbial community structure and the relationship with soil carbon and nitrogen in an original Korean pine forest of Changbai Mountain, China. BMC Microbiol. 19:218. doi: 10.1186/s12866-019-1584-6, PMID: 31519147 PMC6743161

[ref45] Liu L. Zhu K. Krause S. M. B. Li S. P. Wang X. Zhang Z. C.. (2021). Changes in assembly processes of soil microbial communities during secondary succession in two subtropical forests. Soil Boil. Biochem. 154:108144. doi: 10.1016/j.soilbio.2021.108144

[ref46] Luan L. Liang C. Chen L. J. Wang H. T. Xu Q. S. Jiang Y. J.. (2020). Coupling bacterial community assembly to microbial metabolism across soil profiles. mSystems. 5:e00298-20. doi: 10.1128/mSystems.00298-20, PMID: 32518195 PMC7289589

[ref47] Luo S. Yin J. Peng Y. Xie J. Wu H. T. He D. L.. (2020). Glutathione is involved in detoxification of peroxide and root nodule symbiosis of *Mesorhizobium huakuii*. Curr. Microbiol. 77, 1–10. doi: 10.1007/s00284-019-01784-8, PMID: 31624868

[ref400] Magoč T. Salzberg S. L. (2011). FLASH: fast length adjustment of short reads to improve genome assemblies. Bioinformatics 27, 2957–2963. doi: 10.1093/bioinformatics/btr507 21903629 PMC3198573

[ref48] Massana R. Logares R. (2013). Eukaryotic versus prokaryotic marine picoplankton ecology. Environ. Microbiol. 15, 1254–1261. doi: 10.1111/1462-2920.12043, PMID: 23206217

[ref49] Mueller K. E. Hobbie S. E. Chorover J. Reich P. B. Eisenhauer N. Castellano M. J.. (2015). Effects of litter traits, soil biota, and soil chemistry on soil carbon stocks at a common garden with 14 tree species. Biogeochemistry 123, 313–327. doi: 10.1007/s10533-015-0083-6

[ref50] Myers J. A. Chase J. M. Jiménez I. Jørgensen P. M. Araujo-Murakami A. Paniagua-Zambrana N.. (2013). Beta-diversity in temperate and tropical forests reflects dissimilar mechanisms of community assembly. Ecol. Lett. 16, 151–157. doi: 10.1111/ele.12021, PMID: 23113954

[ref51] Olesen J. M. Bascompte J. Dupont Y. L. Jordano P. (2007). The modularity of pollination networks. Proc. Natl. Acad. Sci. U. S. A. 104, 19891–19896. doi: 10.1073/pnas.0706375104, PMID: 18056808 PMC2148393

[ref52] Pan Y. Q. Kang P. Hu J. P. Song N. P. (2021). Bacterial community demonstrates stronger network connectivity than fungal community in desert-grassland salt marsh. Sci. Total Environ. 798:149118. doi: 10.1016/j.scitotenv.2021.149118, PMID: 34332392

[ref53] Pang Y. Tian J. Zhao X. Chao Z. Wang Y. C. Zhang X. P.. (2020). The linkages of plant, litter and soil C:N:P stoichiometry and nutrient stock in different secondary mixed forest types in the Qinling Mountains, China. Peer J. 8:e9274. doi: 10.7717/peerj.9274, PMID: 32547880 PMC7275688

[ref54] Prober S. M. Leff J. W. Bates S. T. Borer E. T. Firn J. Harpole W. S.. (2015). Plant diversity predicts beta but not alpha diversity of soil microbes across grasslands worldwide. Ecol. Lett. 18, 85–95. doi: 10.1111/ele.12381 25430889

[ref55] Qu Z. L. Liu B. Ma Y. Xu J. Sun H. (2020). The response of the soil bacterial community and function to forest succession caused by forest disease. Funct. Ecol. 34, 2548–2559. doi: 10.1111/1365-2435.13665

[ref56] Quast C. Pruesse E. Yilmaz P. Gerken J. Schweer T. Yarza P.. (2013). The SILVA ribosomal RNA gene database project: improved data processing and web-based tools. Nucleic Acids Res. 41, D590–D596. doi: 10.1093/nar/gks1219, PMID: 23193283 PMC3531112

[ref57] Rousk J. Bååth E. Brookes P. C. Lauber C. L. Lozupone C. Caporaso J. G.. (2010). Soil bacterial and fungal communities across a pH gradient in an arable soil. ISME J. 4, 1340–1351. doi: 10.1038/ismej.2010.58, PMID: 20445636

[ref500] Schmidt V. Plenz B. Pfaff M. Pees M. (2012). Disseminated systemic mycosis in Veiled chameleons (Chamaeleo calyptratus) caused by Chamaeleomyces granulomatis. Vet. Microbiol. 161, 145–154. doi: 10.1016/j.vetmic.2012.07.017, PMID: 22857978

[ref58] Sheng Y. Cong J. Lu H. Yang L. Liu Q. Li D.. (2019). Broad-leaved forest types affect soil fungal community structure and soil organic carbon contents. Microbiol. Open. 8:e874. doi: 10.1002/mbo3.874, PMID: 31215766 PMC6813455

[ref59] Shi S. Nuccio E. E. Shi Z. J. He Z. Zhou J. Firestone M. K. (2016). The interconnected rhizosphere: high network complexity dominates rhizosphere assemblages. Ecol. Lett. 19, 926–936. doi: 10.1111/ele.12630, PMID: 27264635

[ref60] Siles J. A. Margesin R. (2016). Abundance and diversity of bacterial, archaeal, and fungal communities along an altitudinal gradient in alpine forest soils: what are the driving factors? Microb. Ecol. 72, 207–220. doi: 10.1007/s00248-016-0748-2, PMID: 26961712 PMC4902835

[ref61] Šnajdr J. Valášková V. Merhautová V. Cajthaml T. Baldrian P. (2008). Activity and spatial distribution of lignocellulose-degrading enzymes during forest soil colonization by saprotrophic basidiomycetes. Enzym. Microb. Technol. 43, 186–192. doi: 10.1016/j.enzmictec.2007.11.008

[ref62] Starke R. Mondéjar R. L. Human Z. R. Navrátilová D. Štursová M. Větrovský T.. (2021). Niche differentiation of bacteria and fungi in carbon and nitrogen cycling of different habitats in a temperate coniferous forest: a metaproteomic approach. Soil Biol. Biochem. 155:108170. doi: 10.1016/j.soilbio.2021.108170

[ref63] Stegen J. C. Lin X. Fredrickson J. K. Chen X. Kennedy D. W. Murray C. J.. (2013). Quantifying community assembly processes and identifying features that impose them. ISME J. 7, 2069–2079. doi: 10.1038/ismej.2013.93, PMID: 23739053 PMC3806266

[ref64] Stegen J. C. Lin X. J. Fredrickson J. K. Konopka A. E. (2015). Estimating and mapping ecological processes influencing microbial community assembly. Front. Microbiol. 6:370. doi: 10.3389/fmicb.2015.00370, PMID: 25983725 PMC4416444

[ref65] Sul W. J. Asuming-Brempong S. Wang Q. Tourlousse D. M. Penton C. R. Deng Y.. (2013). Tropical agricultural land management influences on soil microbial communities through its effect on soil organic carbon. Soil Biol. Biochem. 65, 33–38. doi: 10.1016/j.soilbio.2013.05.007

[ref66] Sun S. Li S. Avera B. N. Strahm B. D. Badgley B. D. (2017). Soil bacterial and fungal communities show distinct recovery patterns during forest ecosystem restoration. Appl. Environ. Microbiol. 83, e00966–e00917. doi: 10.1128/aem.00966-17 28476769 PMC5494632

[ref67] Tian J. Qiao Y. Wu B. Chen H. Li W. Jiang N.. (2017). Ecological succession pattern of fungal community in soil along a retreating glacier. Front. Microbiol. 8:1028. doi: 10.3389/fmicb.2017.01028, PMID: 28649234 PMC5465267

[ref68] Tripathi B. M. Edwards D. P. Mendes L. W. Kim M. Dong K. Kim H.. (2016). The impact of tropical forest logging and oil palm agriculture on the soil microbiome. Mol. Ecol. 25, 2244–2257. doi: 10.1111/mec.13620, PMID: 26994316

[ref69] Tripathi B. M. Stegen J. C. Kim M. Dong K. Adams J. M. Lee Y. K. (2018). Soil pH mediates the balance between stochastic and deterministic assembly of bacteria. ISME J. 12, 1072–1083. doi: 10.1038/s41396-018-0082-4, PMID: 29515169 PMC5864241

[ref70] Wang W. Akhtar K. Ren G. Yang G. Feng Y. Yuan L. (2019). Impact of straw management on seasonal soil carbon dioxide emissions, soil water content, and temperature in a semi-arid region of China. Sci. Total Environ. 652, 471–482. doi: 10.1016/j.scitotenv.2018.10.207, PMID: 30368177

[ref71] Wang X. B. Lu X. T. Yao J. Wang Z. W. Deng Y. Cheng W. X.. (2017). Habitat-specific patterns and drivers of bacterial beta-diversity in China's drylands. ISME J. 11, 1345–1358. doi: 10.1038/ismej.2017.11, PMID: 28282041 PMC5437346

[ref72] Wang K. F. Qiu Z. L. Zhang M. Li X. Y. Fang X. Zhao M. Y.. (2022). Effect of afforestation mode on rhizosphere soil physicochemical properties and bacterial community structure of two major tree species in Xiong’an new area. For. Ecol. Manag. 520:120361. doi: 10.1016/j.foreco.2022.120361

[ref73] Wang Y. Zhang R. Zheng Q. Deng Y. van Nostrand J. D. Zhou J.. (2016). Bacterioplankton community resilience to ocean acidification: evidence from microbial network analysis. ICES J. Mar. Sci. 73:865875, 865–875. doi: 10.1093/icesjms/fsv187

[ref74] Xiong J. Sun H. Peng F. Zhang H. Xue X. Gibbons S. M.. (2014). Characterizing changes in soil bacterial community structure in response to short-term warming. FEMS Microbiol. Ecol. 89, 281–292. doi: 10.1111/1574-6941.12289, PMID: 24476229

[ref75] Xu D. D. Ling J. F. Qiao F. Xi P. G. Zeng Y. N. Zhang J. F.. (2022). Organic mulch can suppress litchi downy blight through modification of soil microbial community structure and functional potentials. BMC Microbiol. 22:155. doi: 10.1186/s12866-022-02492-3, PMID: 35689202 PMC9188084

[ref76] Xue L. Ren H. D. Li S. Leng X. H. Yao X. H. (2017). Soil bacterial community structure and co-occurrence pattern during vegetation restoration in karst rocky desertification area. Front. Microbiol. 8:2377. doi: 10.3389/fmicb.2017.02377, PMID: 29250053 PMC5717032

[ref77] Yang H. (2017). China’s natural forest protection program: progress and impacts. For. Chron. 93, 113–117. doi: 10.5558/tfc2017-017

[ref78] Yang K. Shi W. Zhu J. J. (2013). The impact of secondary forests conversion into larch plantations on soil chemical and microbiological properties. Plant Soil 368, 535–546. doi: 10.1007/s11104-012-1535-6

[ref79] Yao M. Rui J. Li J. Wang J. Cao W. Li X. (2018). Soil bacterial community shifts driven by restoration time and steppe types in the degraded steppe of Inner Mongolia. Catena 165, 228–236. doi: 10.1016/j.catena.2018.02.006

[ref80] Yao M. Rui J. Niu H. Heděnec P. Li J. He Z.. (2017). The differentiation of soil bacterial communities along a precipitation and temperature gradient in the eastern Inner Mongolia steppe. Catena 152, 47–56. doi: 10.1016/j.catena.2017.01.007

[ref81] You Y. Wang J. Huang X. Tang Z. Liu S. Sun O. J. (2014). Relating microbial community structure to functioning in forest soil organic carbon transformation and turnover. Ecol. Evol. 4, 633–647. doi: 10.1002/ece3.969, PMID: 25035803 PMC4098142

[ref82] Zhang Y. Peng C. Li W. Tian L. Zhu Q. Chen H.. (2016). Multiple afforestation programs accelerate the greenness in the ‘three north’ region of China from 1982 to 2013. Ecol. Indic. 61, 404–412. doi: 10.1016/j.ecolind.2015.09.041

[ref600] Zhou J. Liu W. Deng Y. Jiang Y. H. Xue K. He Z.. (2013). Stochastic assembly leads to alternative communities with distinct functions in a bioreactor microbial community. mBio 4. doi: 10.1128/mBio.00584-12 PMC3585448 23462114

[ref83] Zhou J. Ning D. (2017). Stochastic community assembly: does it matter in microbial ecology? Microbiol. Mol. Biol. Rev. 81, e00002–e00017. doi: 10.1128/mmbr.00002-17 29021219 PMC5706748

[ref84] Zhu B. Li C. Wang J. Li J. Li X. (2020). Elevation rather than season determines the assembly and co-occurrence patterns of soil bacterial communities in forest ecosystems of mount Gongga. Appl. Microbiol. Biotechnol. 104, 7589–7602. doi: 10.1007/s00253-020-10783-w, PMID: 32686007

[ref85] Zhu J. J. Liu Z. G. Wang H. X. Yan Q. L. Fang H. Y. Hu L. L.. (2008). Effects of site preparation on emergence and early establishment of *Larix olgensis* in montane regions of northeastern China. New For. 36, 247–260. doi: 10.1007/s11056-008-9097-4

[ref86] Ziegler M. Seneca F. O. Yum L. K. Palumbi S. R. Voolstra C. R. (2017). Bacterial community dynamics are linked to patterns of coral heat tolerance. Nat. Commun. 8:14213. doi: 10.1038/ncomms14213, PMID: 28186132 PMC5309854

