# Supplementary material for: A comparison of microbial composition under three tree ecosystems using the stochastic process and network complexity approaches
Source: Front Microbiol. 2022 Oct 10;13:1018077. doi: 10.3389/fmicb.2022.1018077 (PMC9589112; doi:10.3389/fmicb.2022.1018077)
Supplement: Supplementary file 1 [file Data_Sheet_1.docx]

**Supplementary Figure 1** The fit of the neutral community model (NCM) of bacterial (A) and fungal (B) community assembly under forest stand differences. Solid lines indicate the best fit to the NCM, and the dashed lines represent 95% confidence intervals around the model prediction. OTUs that occur more or less frequently than predicted by the NCM are shown in different colors. Nm indicates the metacommunity size times immigration, R^2^ indicates the fit to this model.


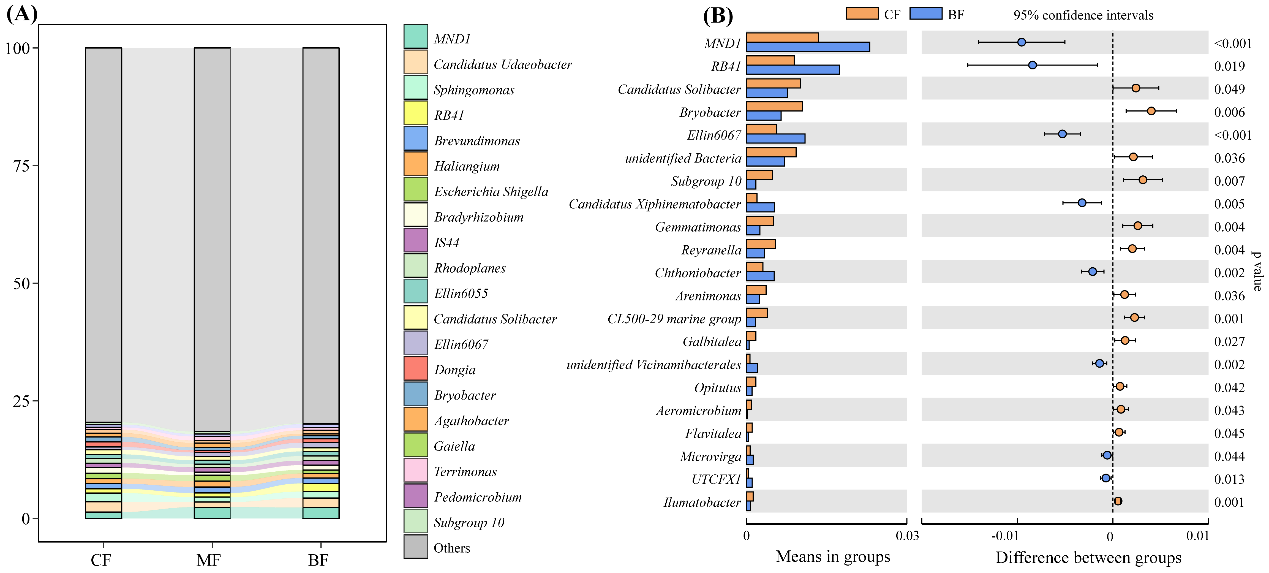


**Supplementary Figure 2** Relative abundance (top 20) at the genera level in the bacterial (A) communities and the significantly different in bacterial (B) genera between CF and BF plots.


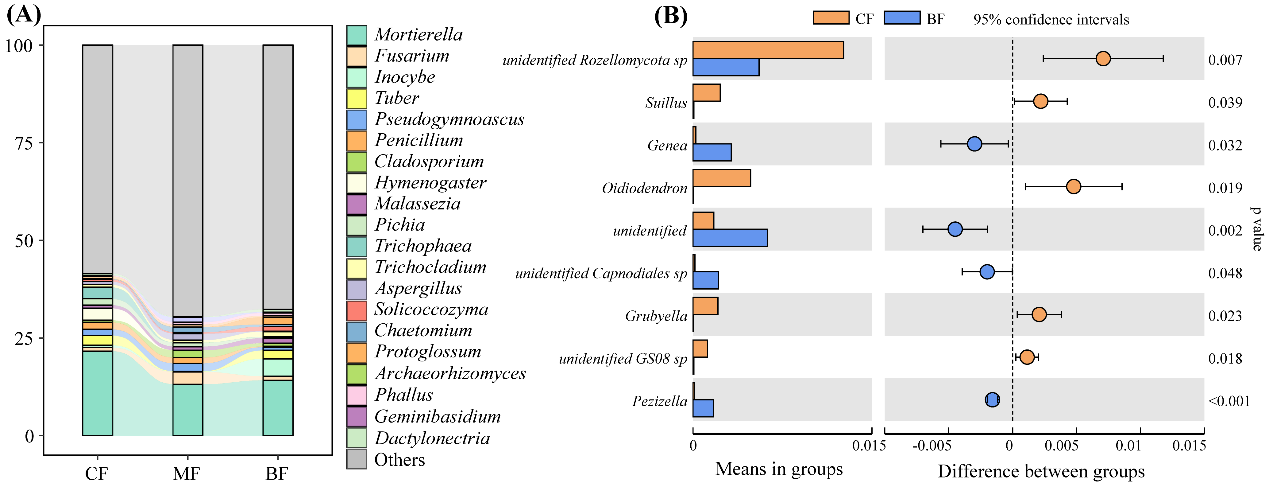


**Supplementary Figure 3** Relative abundance (top 20) at the genera level in the fungal (A) communities and the significantly different in fungal (B) genera between CF and BF plots.

**Supplementary Figure 4** OTU with the top 20 degree in bacterial (A) and fungal (B) co-occurrence networks under forest stand differences.
